# Health financing strategies to reduce out-of-pocket burden in India: a comparative study of three states

**DOI:** 10.1186/s12913-018-3633-5

**Published:** 2018-11-03

**Authors:** Montu Bose, Arijita Dutta

**Affiliations:** 1 Department of Business & Sustainability, TERI School of Advanced Studies, New Delhi, India; 2 Department of Economics, University of Calcutta, Kolkata, India

**Keywords:** Health financing, Fair Price medicine shop, Benefit incidence analysis, Health equity, Out-of-pocket health expenditure, Utilization of public in-patient care, H22, I14, I15, I18

## Abstract

**Background:**

To achieve the Sustainable Development Goals, Indian States have implemented different strategies to arrest high out-of-pocket expenditure (OOPE) and to increase equity into healthcare system. Tamil Nadu (TN) and Rajasthan have implemented free medicine scheme in all public hospitals and West Bengal (WB) has devised Fair Price Medicine Shop (FPMS) scheme, a public-private-partnership model in the state. In this background, the objectives of the paper are to -

1. Study the utilization pattern of public in-patient care facilities for the states,
2. Examine the effectiveness of the strategies adopted by the states to arrest high OOPE and
3. Analyze the extent of equity in public in-patient care services in the states.

**Methods:**

National Sample Survey (71st and 60th round) data, Detailed Demand for Grants of the state governments and the National Rural/Urban Health Mission data have been used for the study. Exploratory data analysis and benefit incidence analysis have been applied to estimate the utilization, OOPE and extend of equity in the states.

**Results:**

The results show that overall utilization of public facilities in TN and Rajasthan has increased substantially; whereas, utilization of public facility has decreased in WB even among the poorest. In addition, OOPE for both medical and medicine is the highest in WB among three states for public sector hospitalizations. Surprisingly, OOPE on medicine is the highest for the poorest class of WB. Analysis showed that the mismatch between actual need and FPMS drug-list has led to high OOPE in the state. Overall, benefit incidence of public subsidy is the highest among the poorest class in all the states. However, geographical sector-wise inequity in public subsidy distribution persists in the states. Analysis of cost of inpatient care shows that TN provides the maximum subsidy for hospitalization and WB provides the minimum. An inverse relationship between utilization of inpatient care and public subsidy has been observed from the analysis.

**Conclusion:**

In conclusion we could say that TN & Rajasthan have successfully implemented their health financing strategies to reduce the health expenditure burden. However, policy-level changes are required to improve the situation in WB.

**Electronic supplementary material:**

The online version of this article (10.1186/s12913-018-3633-5) contains supplementary material, which is available to authorized users.

## Background

Following the ideas of fairness and justice, it is normally accepted that the distribution of health care, often recognized as a merit good, should be determined by the people’s need and not by ability to pay. Since health care contributes to a person’s basic capability to function [1], juxtaposed by the large randomness in occurrence of ill-health, inequalities in health care provisioning might aggravate inequalities in people’s capability to function and hence in all non-health attributes [2]. Therefore, to protect people’s capability to function, the reduction of inequalities in health care is primarily aimed at. The social planner, very often the government, thus targets to allocate resources not to maximize the total gains in health, but to provide a more equal distribution of health benefits. In this sense, not just the equal treatment for the equals (horizontal equity) is targeted but allows for unequal treatment to unequals (vertical equity) [3]. However, in most of the developing countries including India, many people do not have access to health facilities at all, while majority with access to healthcare facilities face a monumentous financial pressure to meet the healthcare expenditure. In such situation, supportive roles of the government in providing and financing health care assumes crucial importance to arrest such financial burden and offer a cushion to households to avoid the medical poverty trap. It is generally accepted that government health expenditures should disproportionately benefit the poor. And yet in most developing countries the opposite is the case and the subsidy benefit is enjoyed disproportionately more by the richer class [4–7].

National Health Account estimates for India 2013–14 [3] shows that government spending on health is only 1.15% of the Gross Domestic Product (GDP) of India and the current health expenditure is even lower for the country. Low level of investment in health by the government forces people to spend a larger share of health care expenditure from their pocket for utilization of the services. It has been found that about 4.5% people are impoverished due to high out-of-pocket (OOP) expenditure [8] in India. It has also been documented that expenditure on medicine constitutes about 70% of the total out-of-pocket expenditure in India [9–11]. In these circumstances, to achieve the Universal Health Coverage (UHC), Indian states have implemented different strategies to arrest high OOP expenditure and to increase equity into healthcare system. To strengthen the health system and to achieve universal access to affordable, equitable and quality health care services, both state and the central governments have taken certain initiatives under National Health Mission since 2005. This program, as well as the state-specific programs related to medicines are all universal programs, aiming to cover all citizens of India. On the other hand, to provide financial protection against high OOP expenditure and to improve the access to quality healthcare services National Health Insurance Scheme (Rashtriya Swasthya Bima Yojana or RSBY^1^) has been introduced since April 2008. Initially, the scheme was targeted only to the below poverty line (BPL) households. However, later the programme was extended to cover some defined unorganized sector workers.^2^ It has to be noted that Tamil Nadu has not implemented the RSBY scheme. Tamil Nadu provides insurance coverage through Chief Minister’s Comprehensive Health Insurance Scheme (CMCHIS) to the people who have annual income less than INR 72,000. Similarly, Tamil Nadu implemented free medicine distribution scheme for all who are utilizing public health facilities for treatment since 1995. Following Tamil Nadu (TN), Rajasthan (RAJ) has also implemented similar strategy to distribute free medicine through public health facilities. However, West Bengal (WB) has implemented a public-private partnership (PPP) model to provide medicine at a very high discount (generic medicine) for all (who have a valid prescription) through fair-price medicine shop (FPMS^3^) located within public hospital premises since 2012. At the onset, it appears that the West Bengal model is poised to reduce OOP expenditures of patients in both public and private facilities (as people from any facility could purchase the medicine from the fair price medicine shop), TN & RAJ models would support those who opted for treatment in public health facilities. If we look from the government’s perspective we could see that, government has to make a considerable amount of spending to run the programme in both TN and RAJ, though in West Bengal, government played the role of a regulator only; there is no financial burden on the government associated with the model.

In this background, the present study attempts to critically examine the effectiveness of the policies in achieving the Sustainable Development Goals (SDGs). Specifically, the SDG-3 emphasizes on ‘financial risk protection, access to quality essential health-care services and access to safe, effective, quality and affordable essential medicines and vaccines for all’ to achieve Universal Health Coverage by 2030 [12]. Therefore, in this study an attempt will be made to –

- Study the utilization pattern of public in-patient care facilities for the three states,
- Examine the effectiveness of the strategies adopted by the states to arrest high OOP expenditure and
- Analyze the extent of equity in public in-patient care services in the states.

Specifically, effectiveness of the financing strategies implemented by the states would be reflected on utilization of public healthcare facilities for treatment, OOPE for hospitalization and equity in distribution of public subsidy. Available literature has already documented affordability as one of the major barriers to access to healthcare services [13–17]. The major target of all the schemes (RSBY, CMCHIS and respective medicine distribution schemes in individual states) is to increase utilization of public healthcare facilities by removing the barrier of affordability. As the schemes on access to drugs aim to provide listed essential medicines either for free (in TN & RAJ) or at discounted prices (in WB), they are expected to reduce the total OOPE for hospitalization, thus offering financial protection for the hospitalized. This framework of health system research thus targets to identify the journey of three Indian states in terms of access, financial risk protection and equity in subsidy distribution of Indian population, particularly of the more vulnerable section.

The paper has been divided into five sections. Background section provides a brief background and the objectives of the paper, data sources and the methodology of the paper is discussed in the Methods section. Results section provides the results of the paper, Discussion section discusses the results and the final Section concludes the paper.

## Methods

National Sample Survey (NSS) 71st round unit level data (2014) on *Social Consumption: Health*, NSS 60th round (2004^4^) unit level data on *Morbidity and Health Care*, Detailed Demand for Grants (DDGs) of the State Governments 2015–16,^5^ National Rural Health Mission (NRHM) Programme Implementation Plan (PIP) data of 2013–14 have been used for the present study. This dataset was collected by household survey method, with the sample frame representative at regional, state and national level. NSS adopted the stratified multi-stage sampling method to collect data for both the rounds. The census villages in the rural sector and the Urban Frame Survey (UFS) blocks in the urban sector were considered as the First Stage Units (FSU). FSUs were selected by Probability Proportion to Size^6^ with Replacement (PPSWR) in both the sectors. Households of both the sectors were considered as the Ultimate Stage Units (USU) to collect information from all Indian States and Union Territories (UTs). Following the sampling technique, NSS has collected information from 3917 households from TN in 71st round, 2912 households in Rajasthan and 5019 households from WB. The corresponding sample households for the 60th round were 5139, 3383 and 5049 for TN, Rajasthan and WB respectively. The data reports household level information as well as the details of the individuals. It also records the details of morbidity and hospitalization of the individuals along with the level of care (sub-center, primary health center, community health center etc.), type of facilities (public or private, hospital or dispensary etc.) and corresponding out-of-pocket expenditure for consultation, medicine, diagnostic tests and other related services. Information on insurance coverage and sources of finance for treatment is also available from the data. Data on morbidity and hospitalization were collected from each member of the households. Recall period for hospitalization was 365 days in both the rounds.

To estimate the utilization of publicly provided healthcare services and the OOPE, descriptive statistical methods have been applied. To represent the utilization of public sector hospitals (U_pub_), percentage share of public sector hospitalization (say, H_pub_) in total hospitalization (say, H_pub + pvt_) cases has been calculated. Mathematically, Upub = $\frac{H_{pub}}{H_{pub+ pvt}}\ X\ 100$. The (arithmetic) mean out-of-pocket expenditure for hospitalization cases has been used as an estimator of per episode OOPE (medical or medicine) for hospitalization. The distribution of subsidy benefit among various socio-economic groups has been estimated by the Benefit Incidence Analysis (BIA). To estimate the benefit incidence, information is needed on the share of a particular socio-economic group^7^ (say j) in the utilization of a service (say i) and the government’s net expenditure (or subsidy) on the service i (here in-patient care). Present study followed the methodology available from different studies [14–17]. In short, the product of the net subsidy for a publicly provided service (here in-patient care) and the utilization share of a socio-economic class (here MPCE class – say poor, middle class rich etc.) in total utilization of the service would give us the share of subsidy benefit for that particular group.

Mathematically, the Benefit Incidence is estimated (detail is provided at Additional file 1) by the formula –.

$$ {\psi}_j=\sum {\varepsilon}_{ij}\frac{\mu }{\varepsilon_i}=\sum {\chi}_{ij}{\mu}_i $$

Where,

*ψ*_*j*_ = Benefit of public subsidy enjoyed by group j (here MPCE class); *ε*_*ij*_ = utilization of service i by group j; *ε*_*i*_ = utilization of service i by all groups together; *μ*_*i*_ = government’s net expenditure on service i and *χ*_*ij*_ = group j’s share of utilization of service i.

To cross-validate the estimates, another set of analysis has been made for each state using government budget data. To get the information on total public expenditure on health we have used the state specific Detailed Demand for Grants (DDGs) of 2015–16 [18–20]. The 2013–14 actual expenditure on health by the Ministry of Health and Family Welfare is available from 2015 to 16 State Expenditure Budget document (DDGs). Each line item^8^ of the DDGs has been cross-classified for health care functions (HC^9^) following the System of Health Accounts (SHA 2011) guideline [21]. Similar exercise has been carried out to cross-classify each expenditure head of the NHM PIP^10^ (2013–14) to identify the expenditure on inpatient curative care [22]. Then we have estimated the total public expenditure on in-patient curative care through Expenditure Budget (Ministry of Health and Family Welfare) and NHM.^11^

Following Srivastava et al., 2016; Bose & Dutta 2015; Bose 2014 [14–16], we have considered the out-of-pocket (OOP) expenditure at private hospital as the proxy of the actual cost of providing the service at the public hospital. To estimate the subsidy, we have followed the below steps –

- We have calculated the modal value of the OOP expenditure in private hospitals (α_pq_) for each disease in the rural and urban sector separately for in-patient care service (where p = disease and q = sector),
- The modal private OOP expenditure amount (α_pq_) has been weighted with the number of patients (β_pq_) in each disease category of a particular region (rural/urban), utilizing public health facilities for in-patient care treatment (say, ρ_pq_ = α_pq_ x β_pq_).
- The share (ρ_pq_/∑ρ_pq_) of each category (combining disease and region) in total expenditure (∑ρ_pq_) has been multiplied with the total public expenditure (Ω) to get the disease specific subsidy for each sector (Г_pq_).

To compare the cost of services in the public hospitals of the states, we have followed the *cost of care* methodology^12^ available from Tamil Nadu State Health Accounts 2013–14 [8]. To study the effectiveness of financing strategies the results of 2014 have been compared with the estimates of 2004.

## Results

### Utilization

Increase in utilization of health care facilities for treatment is an important objective of any health care system. Especially, increase in utilization of public facilities would help to achieve the health goals by reducing out-of-pocket expenditure. Analyzing the NSS data on morbidity and health care for 60th (2004) and 71st (2014) round, it has been observed that hospitalization in public hospitals has decreased in West Bengal during the period 2004–2014 (Table 1). On the other hand, Rajasthan is experiencing a sharp rise in utilization of public health care facilities. Utilization of public hospitals for in-patient care in Tamil Nadu remains stagnant during this time period. Utilization of urban public hospital in 2014 recorded a drop in all the three states considered when compared to 2004, with urban West Bengal experiencing the steepest decline. Contrary to this, utilization of the rural sector of West Bengal is the highest among the three states (77.5%). On the other hand, public sector utilization in the rural sector for both Tamil Nadu and Rajasthan has increased during this period.

**Table 1:** Share of Public Sector in Total Hospitalization of the States (in %)

Year	TN			RAJ			WB		
	R	U	C	R	U	C	R	U	C
2004	40.2	36.2	38.8	52.1	63.4	55.2	78.6	65.6	74.3
2014	45.4	32.6	38.9	65.6	58.1	63.6	77.5	55.1	70.4

Note: R-Rural, U-Urban, C-Combined

Source: Authors’ estimation from NSS 60th and 71st round data

Table 2 reports the utilization of public facilities across MPCE classes for both the time periods. It has been observed that during 2004, poorest class of West Bengal had the highest utilization share (32.69%). At that time, utilization share of the poorest class in Tamil Nadu and Rajasthan were 30.52% & 30.78% respectively. However, in 2014, while the share of the poorest class has dropped to 29.72% in West Bengal, the corresponding shares increased substantially in Tamil Nadu (41.34%) and Rajasthan (37.14%).

**Table 2:** MPCE Class wise Utilization Share of Public Facilities for Inpatient Care (in %)

Sector	MPCE	TN		RAJ		WB	
		2004	2014	2004	2014	2004	2014
Rural	P	22.30	*35.28*	27.41	*37.51*	*28.76*	*28.45*
	LM	*33.39*	26.43	*31.58*	21.30	25.65	22.80
	UM	26.08	23.73	14.39	22.72	25.15	27.03
	R	18.23	14.56	26.62	18.47	20.44	21.73
Urban	P	*47.63*	*49.42*	*38.38*	*35.94*	*42.13*	*33.56*
	LM	23.60	18.84	21.84	32.52	35.74	32.87
	UM	21.51	22.77	18.13	19.38	10.99	23.50
	R	7.26	8.96	21.65	12.16	11.14	10.06
Combine	P	*30.52*	*41.34*	*30.78*	*37.14*	*32.69*	*29.72*
	LM	30.21	23.18	28.59	23.99	28.61	25.29
	UM	24.60	23.32	15.54	21.92	20.99	26.16
	R	14.67	12.16	25.09	16.95	17.71	18.84

Note: P-poorest, LM-lower middle, UM-upper middle, R-richest

Source: Authors’ estimation from unit level NSS 60th and 71st round data

The italics numbers in the tables signify the maximum

Analyzing the data separately for rural and urban sector we could see that, utilization of the poorest class in rural area in West Bengal remained stagnant, though the opposite is true for other two states (from 22.3 to 35.3% and in Tamil Nadu and from 27.4 to 37.5% in Rajasthan respectively). On the other hand, utilization share has decreased for the urban poorest class in both West Bengal and Rajasthan, though the decrease is sharper in the former state. Contrary to this, utilization share of the urban poorest class of Tamil Nadu has increased during the period. NSS also provides information on utilization of various services (like surgery, medicine, diagnostic tests etc.) during hospitalization. Analyzing this information for public sector hospitalization, it is observed that utilization of all the services has increased substantially for the Poor class in TN and RAJ (Additional file 2: Table A5). However, in WB, except surgery and other diagnostic tests, Poor class is experiencing a sharp decrease in utilization of these services when compared to 2004.

### Out-of-pocket expenditure

Almost all the public policies in the health sector have a direct or indirect goal to reduce out-of-pocket expenditure; therefore, it is worthy to study the OOP expenditure scenario of the states in the two-time periods. In Table 3 we have presented the OOP expenditure for medicine and for total medical treatment separately. For better comparability, we have reported the expenditure of 2004 and 2014 separately for public and private sector hospitalization.

**Table 3:** Per-episode Out-of-pocket Expenditure during Hospitalization (in INR)

State	Expenditure on Medicine				Medical Expenditure			
	2004		2014		2004		2014	
	Public	Private	Public	Private	Public	Private	Public	Private
*TN*	102.41	1125.90	150.16	*3920.06*	1391.69	11766.71	450.85	*19264.71*
*RAJ*	*1725.07*	*2228.25*	1516.13	3451.26	*6212.58*	10691.45	3628.62	22946.43
*WB*	1326.12	1934.32	*1916.52*	2816.03	3222.24	*13715.03*	*5602.78*	17951.06

Note: 2014 prices are converted into 2004 prices; Medical expenditure also includes medicine prices

Source: Authors’ estimation based on NSS 60th and 71st round data

The italics numbers in the tables signify the maximum

It has been observed that, in 2004, OOP expenditure on medicine during hospitalization in public hospitals was the maximum in Rajasthan (INR. 1725) followed by West Bengal (INR. 1326). Tamil Nadu recorded a far lower OOP expenditure, in the tune of less than 10% of the corresponding figures in other two states (INR. 102). However, in 2014, the OOP expenditure recorded the maximum level for West Bengal (INR. 1917) followed by Rajasthan (INR. 1516). The OOP expenditure on medicine during public sector hospitalization is the lowest in Tamil Nadu (INR. 150) during 2014 also. It has to be noted that, the OOP expenditure on medicine during private sector hospitalization is the lowest in West Bengal (INR. 2816) among three states in 2014. Total medical expenditure in the public institutions, on the other hand, was the maximum for Rajasthan (INR. 6213) in 2004 followed by West Bengal (INR. 3222) and Tamil Nadu (INR. 1392). In 2014, the corresponding expenditure becomes the highest for West Bengal (INR. 5603) followed by Rajasthan (INR. 3629) and Tamil Nadu (INR. 451). Importantly, overall OOP medical expenditure at the private sector was the highest in West Bengal (INR. 13,715) during 2004. However, in 2014, Rajasthan (INR. 22,946) experiences the highest OOP medical expenditure followed by Tamil Nadu (INR. 19,265) for private sector hospitalization. The private OOP medical expenditure becomes the lowest for West Bengal (INR. 17,951) during this period.

If we look at the per episode OOP expenditure on medicine by different MPCE classes during 2014, we could see that the poorest class (INR. 3438) spends the maximum amount for hospitalization in public sector compared to other MPCE groups in West Bengal (Table 4). Whereas, in case of private sector hospitalization, the richest class (INR. 5371) has the highest OOP expenditure for purchasing medicine followed by the poorest class (INR. 3576) in the state. For the other two states – Tamil Nadu and Rajasthan – the richest class has the highest and the poorest class has the lowest OOP medicine expenditure in both public and private sector hospitalization.

**Table 4:** MPCE Class wise per-episode OOP Expenditure on Medicine in 2014 (in INR)

MPCE Class	TN		RAJ		WB	
	Public	Private	Public	Private	Public	Private
P	64.72	3937.05	1264.77	2766.55	*3438.17*	3576.45
LM	72.12	5561.21	2132.33	3836.96	2551.06	2058.38
UM	320.19	3808.41	1641.08	4976.74	1718.48	2862.76
R	*524.40*	*6978.39*	*3549.75*	*5602.23*	2356.55	*5370.90*
All	*196.87*	*5139.63*	*1987.81*	*4524.98*	*2530.77*	*3692.13*

Note: P-poorest, LM-lower middle, UM-upper middle, R-richest

Source: Authors’ estimation based on NSS 71st round data

The italics numbers in the tables signify the maximum

Further analyzing the data for MPCE class and geographical location (rural and urban) wise OOP expenditure on medicine during public sector hospitalization, we could see that, both in Tamil Nadu and Rajasthan the poorest class spends the lowest among all MPCE classes to purchase medicine during public sector hospitalization (in both the sectors).

In West Bengal, on the other hand, poorest class recorded to have the highest expenditure on medicine during public sector hospitalization among all classes in both the sectors (Table 5). It has to be noted that, the overall OOP expenditure on medicine in the urban sector is twice as compared to the rural sector of Tamil Nadu and Rajasthan. However, in West Bengal the difference in overall OOP expenditure on medicine is very low.

**Table 5:** MPCE Class & Sector wise per-episode OOP Expenditure on Medicine at Public Hospital in 2014 (in INR)

MPCE	TN		RAJ		WB	
	Rural	Urban	Rural	Urban	Rural	Urban
P	69.70	60.20	1234.00	1355.00	3749.00	2660.00
LM	60.80	91.70	2166.00	2085.00	2682.00	2373.00
UM	199.50	520.40	1332.00	5038.00	1430.00	2596.00
R	325.40	951.40	2721.00	6209.00	2336.00	2470.00
All	*145.10*	*274.10*	*1741.00*	*3117.00*	*2533.00*	*2527.00*

Note: P-poorest, LM-lower middle, UM-upper middle, R-richest

Source: Authors’ estimation based on NSS 71st round data

The italics numbers in the tables signify the maximum

### Benefit incidence of public subsidy

Benefit Incidence of public subsidy on inpatient care has been computed for both the time periods and are reported in Table 6. It has been observed that during 2004, overall public subsidy was the maximum for the poorest class in Tamil Nadu (41.5%), the richest class in Rajasthan (39.8%) and lower middle class in West Bengal (32.3%). On the other hand, poorest class of all the states has enjoyed the maximum benefit compared to all other MPCE classes during 2014. However, there is a huge inter-state variation in the benefit share. In Tamil Nadu benefit share of the poorest class has increased from 41.5% to 54.2% during this time period. Corresponding benefit share for the poorest class of Rajasthan has increased from 25.4% to 41.5% and in West Bengal it increased only marginally from 30.5% to 31.3%.

**Table 6:** MPCE Class wise Incidence of Public Subsidy (in %)

Sector	MPCE	TN		RAJ		WB	
		2004	2014	2004	2014	2004	2014
Rural	P	20.74	*42.25*	19.28	*44.43*	22.36	25.99
	LM	*36.71*	26.58	*35.72*	15.13	25.96	19.44
	UM	29.21	20.48	16.22	20.66	*29.30*	*28.15*
	R	13.34	10.69	28.78	19.78	22.38	26.41
Urban	P	*57.76*	*64.35*	28.12	38.59	*41.01*	*36.21*
	LM	18.98	11.01	15.69	*38.88*	40.53	31.53
	UM	18.63	20.21	11.30	14.86	6.19	24.66
	R	4.18	4.43	*44.89*	7.66	12.27	7.60
Combine	P	*41.52*	*54.17*	25.35	*41.53*	30.50	*31.30*
	LM	26.87	18.18	21.98	26.94	*32.32*	25.72
	UM	23.36	20.33	12.84	17.78	19.21	26.34
	R	8.25	7.31	*39.83*	13.75	17.96	16.64

Note: P-poorest, LM-lower middle, UM-upper middle, R-richest

Source: Authors’ estimation from NSS 60th and 71st round data

The italics numbers in the tables signify the maximum

In the rural sector, both Tamil Nadu and Rajasthan show that the highest benefit share has shifted from lower middle class to the poorest class during 2004 to 2014. Contrary to this, in West Bengal rural upper middle class continued to gain the maximum benefit of public subsidy. However, benefit share of the rural poorest class of the state has slightly increased from 22.4% to 26.0%. Benefit share of the urban poorest class of Tamil Nadu has increased from 57.8% to 64.4% during this time period. Urban Rajasthan shows almost same benefit share for the poorest and the lower middle class in 2014; however, the share of the poorest class has increased from 28.1% to 38.6%. In West Bengal, the poorest class of urban sector is also getting the maximum share of the public subsidy; however, it has decreased from 41.0% in 2004 to 36.2% in 2014.

To cross-validate our result, we have also analyzed the budget and NHM data of the states. Similar trend of subsidy distribution has been observed from the analysis. However, the subsidy share for the poorest class has been tapered off in the second method,^13^ with difference being marginal in case of West Bengal (see Additional file 2: Table-A3). It has to be noted that, the difference in estimates of the two methods was higher for Tamil Nadu and Rajasthan compared to West Bengal.

**Fig. 1:**
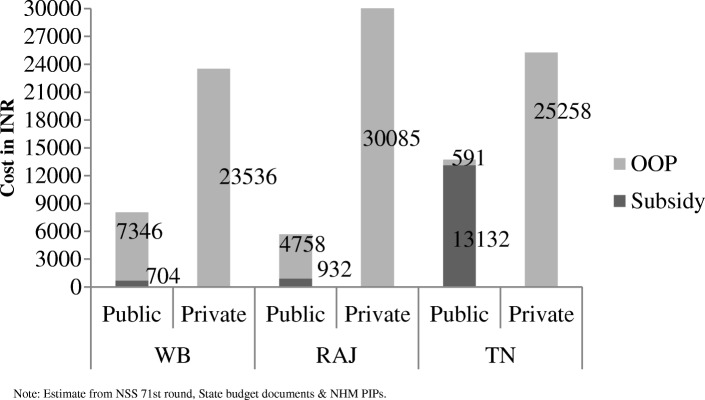
Cost of Providing In-patient Healthcare Facility in Public & Private Facilities in 2014

The analysis of cost of care shows that, treatment cost of a patient in public facility is the lowest in Rajasthan and highest in Tamil Nadu followed by West Bengal. However, more than 95% of the total cost of hospitalization is subsidized in Tamil Nadu (Fig. 1), while the corresponding shares for Rajasthan and West Bengal are 16% and 9% respectively. It has to be noted that West Bengal enjoys a comparative advantage over other two states in private sector hospitalization. The OOP expenditure for private sector hospitalization is the minimum in West Bengal followed by Tamil Nadu and Rajasthan.

## Discussion

Present paper focuses on three Indian states – Tamil Nadu, Rajasthan and West Bengal to study the change in utilization pattern of public health care facilities, impact of different financing strategies on out-of-pocket expenditure during hospitalization and its consequences on equity, which is the ultimate objective of any public health system. It has been observed that overall utilization of public facilities in Tamil Nadu and Rajasthan have increased substantially, whereas, utilization of public facility has decreased in West Bengal during this period. Moreover, while the overall utilization of the poorest class has increased for Tamil Nadu and Rajasthan, that of West Bengal experienced a decline in utilization of public facilities for in-patient care treatment. It is well documented in the literature that patients who utilize public healthcare facilities in West Bengal don’t get other supplementary services (like consultation, diagnostic tests, medicines etc.) as a package [14, 15, 23]. It is also observed from our study that TN & RAJ is providing all healthcare services during public sector hospitalization as a package to the poor patients. However, in WB, the poor patients have limited access to healthcare services during hospitalization. Additionally, it has also revealed from our study that utilization of these healthcare services has decreased substantially for the poor class of the state. This may have induced the poor to stay away from public sector even when needed and rather resort to low quality private health care facilities, often non-institutional under regulatory jargon (Rural medical practitioners, quacks etc.). This forces them to purchase these services from the market and impact of such poor provisioning of services is visible in the utilization of public facilities in the state. This scenario is uniformly observed in both the sectors of the states. Also, the OOP expenditure for both medical and medicine is the highest in West Bengal among three states for public sector hospitalizations, though in 2004 Rajasthan recorded the highest OOP expenditures on medicine. Surprisingly, OOP spending on medicine is the highest for the poorest class of West Bengal, while in Tamil Nadu and Rajasthan, the highest OOP expenditures are incurred by the richest class and as we move towards the poorest class the OOP expenditure on medicine falls.

NSS reports [23, 24] show that access to free medicine and diagnostic tests have increased marginally in West Bengal, but still it’s very low compared to Tamil Nadu and Rajasthan. On the other hand, if we look at the distribution of ailments across MPCE classes in all the states, we could see that non-communicable diseases, injuries and disabilities have increased substantially in all the states (Additional file 2: Table A1). Moreover, most of the people suffering from these diseases prefer to utilize public facilities for their treatment in both the sectors of West Bengal (Additional file 2: Table A2). However, the *mandatory list* of drugs provided by the government (142 medicines) for fair price medicine shop mostly includes medicines related to communicable disease or antibiotics [25]. As a result, most of the people utilizing healthcare facilities are forced to buy medicines from open market and end up with high OOP expenditure on medicine. Overall, benefit incidence of public subsidy is the highest among the poorest class in all the three states.

Also, the paper succinctly brings out that for the poorest and the most marginalized population, the best policy to offer financial protection and access simultaneously is offering free medicine from the hospital pharmacy. With FPMS program in West Bengal, the poor might have received access to medicine more easily, but their OOPE is still positive, while earlier they might have not bought those medicines at all.

However, though the poorest class of Tamil Nadu and Rajasthan can enjoy the highest benefit share in both rural and urban region, rural West Bengal shows the highest benefit share for the upper middle class. High utilization of public facilities and low public investment in health have resulted very low share of subsidy and high OOP expenditure during hospitalization in West Bengal. Rajasthan, on the other hand, is struggling with the high OOP expenditure in the private sector hospitalization. However, following Tamil Nadu, the state has managed to arrest the OOP expenditure in the public-sector hospitalization (Table 7).

**Table 7:** Summary Outcome

Sl. No.	Issues	TN	RAJ	WB
1	Increase in public sector hospitalization	I	Y	N
1.1	Increase in public sector hospitalization in the rural sector	Y	Y	N
1.2	Increase in public sector hospitalization in the urban sector	N	N	N
2	Utilization of public facility become more pro-poor	Y	Y	N
2.1	Utilization of public facility become more pro-poor in the rural sector	Y	Y	I
2.2	Utilization of public facility become more pro-poor in the urban sector	Y	N	N
3	Medical expenditure has increased in the public sector	N	N	Y
4	Medical expenditure has increased in the private sector	Y	Y	Y
5	Medicine expenditure has increased in the public sector	Y	N	Y
6	Medicine expenditure has increased in the private sector	Y	Y	Y
7	Pro-poor OOP medicine expenditure in the public sector	Y	Y	N
8	Pro-poor OOP medicine expenditure in the private sector	N	Y	N
9	Pro-poor OOP medicine expenditure in the rural public hospital	N	Y	N
10	Pro-poor OOP medicine expenditure in the urban public hospital	Y	Y	N
11	Subsidy distribution has turn out to be more pro-poor	Y	Y	Y*
11.1	Subsidy distribution has turn out to be more pro-poor in the rural sector	Y	Y	N
11.2	Subsidy distribution has turn out to be more pro-poor in the urban sector	Y	Y	N

Note: I: remain almost same, Y – Yes & N – No. * it might be surprising that the combined subsidy distribution for WB is pro-poor; however, separate subsidy distribution of the rural and urban sector is not pro-poor. Basically, all the changes are extremely marginal, and it is really very difficult to comment on the move towards or away from pro-poorness after comparing the subsidy amounts only. To get clear picture of the pro-poorness (or richness) of the subsidy distribution, we have calculated the concentration indices of the subsidy distribution and reported in see Additional file 2: Table A4

Therefore, healthcare financing strategies implemented by Tamil Nadu and Rajasthan were fruitful in terms of providing ‘affordable healthcare’ facilities to the financially weaker people. It has helped to increase the utilization of public health care facilities by the desired economic class and to bring equity in the health care system in terms of OOP expenditure and benefit incidence of public subsidies. West Bengal, on the other hand, is experiencing a decrease in utilization of public institutions by the targeted poorest class. Even in terms of OOP expenditure and benefit incidence of the public subsidy the state remains far behind the Southern state – Tamil Nadu. It is clearly observed from the 2004 estimates that, Rajasthan was far behind in terms of all the three indicators (utilization, OOP expenditure and benefit incidence) compared to other two states. However, after successfully implementation of the financing strategies, the state has improved its health system and all the indicators are now better than West Bengal. West Bengal, on the other hand, has failed to arrest the high OOP expenditure for medicines. Even equity in distribution of public subsidy is not persisting in the state. In a nutshell, we could say that, the strategies undertaken by the state has failed to accomplish the desired outcome for it.

The takeaway from this paper for policy analysis is that the best option to create access and financial risk protection simultaneously for the poor is best done by free medicine programs as in TN and Rajasthan. FPMS helped the non-poor more and could not create all the targets under SDG-3 as mentioned in the beginning of the paper.

We should also mention here that, there are some limitations of the present study. We have used NSS data to estimate the utilization pattern, OOP expenditure and benefit incidence of public subsidy. However, as the data is self-reported, there could be some recall bias. To estimate the benefit incidence, we have used the OOP expenditure of the private hospitals as the proxy for cost of providing the same service in the public institutions. However, an appropriate costing study could be helpful to provide more accurate estimate of the subsidy scenario. Due to unavailability of the FMR data, we have used PIP data to capture the expenditure of the government through NHM.

## Conclusion

Summing-up the results we can say that TN model has been successful in achieving its health goals after implementing various health financing strategies. Following the same strategies, RAJ is also reaping the benefit. However, WB has failed to achieve its goals. Focused policies are required to increase public sector utilization of the state. Procuring medicine or regularly updating the essential list of drugs according to need of patients are urgently required in West Bengal to arrest high OOP expenditure on medicine. As the provisions to free other healthcare facilities like diagnostic test etc. are very low in the state, improving access to these services would improve the health scenario of the state. As Tamil Nadu has achieved most of the health goals and poorest class enjoys the public health services the most, the state should now also focus on the lower middle MPCE class for better equity in the system. Rajasthan, on the other hand, after successful implementation of Tamil Nadu model, has improved the health financing indicators of the state drastically. However, the state should focus on the urban poor for better health outcome.

## Additional files

Additional file 1: Illustration of Benefit Incidence Analysis. (DOCX 20 kb)

Additional file 2: Additional tables. (DOCX 59 kb)

## Abbreviations

BIA: Benefit Incidence Analysis
CGHS: Central Government Health Scheme
CMCHIS: Chief Minister’s Comprehensive Health Insurance Scheme
DDG: Detailed Demand for Grant
EDL: Essential Drug List
ESIS: Employment State Insurance Scheme
FMR: Financial Management Report
FPMS: Fair Price Medicine Shop
FSU: First Stage Unit
GDP: Gross Domestic Product
HC: Health Care Functions
INR: Indian Rupee
MGNREGA: Mahatma Gandhi National Rural Employment Guarantee Act.
MPCE: Monthly Per Capita Expenditure
MRP: maximum retail price
NHM: National Health Mission
NRHM: National Rural Health Mission
NSS: National Sample Survey
OOPE: out-of-pocket expenditure
PIP: Programme Implementation Plan
PPP: Public Private Partnership
PPSER: Probability Proportion to Size with Replacement
RAJ: Rajasthan
RSBY: Rashtriya Swasthya Bima Yojana
SDG: Sustainable Development Goal
SHA: System of Health Accounts
TN: Tamil Nadu
UFS: Urban Frame Survey
UHC: Universal Health Coverage
USU: Ultimate Stage Unit
UT: Union Territory
WB: West Bengal

## Footnotes

1 The beneficiaries of RSBY scheme are entitled to get an annual coverage of up to INR 30,000 per household for a listed disease-category related hospitalization. Additionally, transport expenses of INR 100 per hospitalization (with a maximum coverage up to INR 1000 per household) is also covered under the scheme. To enroll in the scheme, households need to pay INR 30 annually as registration charge [26].

2 The unorganized sector workers covered under the scheme are - building and other construction workers registered with the Welfare Boards, licensed railway porters, street vendors, MGNREGA workers who have worked for more than 15 days during the preceding financial year, beedi workers, domestic workers, sanitation workers, mine workers, rickshaw pullers, rag pickers, auto/taxi drive [26].

3 In FPMS model, government invests only land in the public hospital premises and based on the extent of discount on maximum retail price (MRP) private provider gets permission to sell generic medicines to the patients (coming from any facility, public or private). It is mandatory for these FPMS to keep the listed essential drugs (in the government approved EDL) in the shop and according to the contract FPMS are bound to give the agreed discount to all the products they sell from the shop (even other than the EDL medicines).

4 NSS is the only survey available in India which collects information on household characteristics, individual information, all types of morbidity, hospitalizations and related OOPE. NSS conducts such study, only once in a decade. Last two health rounds of survey were conducted by NSS in 2014 and 2004. Therefore, it is not possible to include any analysis in between these two years.

5 The budget document of 2015–16 reports the actual expenditure of 2013–14.

6 The census village as per the latest Census (2011 for 71st round and 2001 for 60th round) for the rural sector and the UFS blocks for the urban sector were treated as the size of the FSUs.

7 For the present study we have considered the economic class only. NSS provides information on monthly expenditure of a household and household size. We have formed the monthly per capita expenditure (MPCE) class from that information separately for rural and urban sector. This MPCE class (poorest, lower middle, upper middle and richest) has been taken as a proxy of the financial status of a household.

8 In the budget document, expenditures are reported at various level and they are categorized as head, major head, sub-major head etc. The broad expenditure categories start from the Head (like Medical & Public Health-2210), major head (like Urban Health Services-Allopathy) and it becomes specific as we move down in the expenditure group. Item head (like house rent allowance, telephone bill, diet, drug, other hospital consumables etc.) is the last layer of the classification.

9 We have analyzed the budget data for health care functions at the two-digit level. Expenditure under various in-patient care (inpatient curative care, inpatient rehabilitative care and inpatient long-term care) has been added together to get the total expenditure on in-patient care.

10 PIP only reports the proposed expenditure budget under each expenditure head. Whereas, Financial Management Report (FMR) provides the actual expenditure under NHM. However, as we don’t have access to FMR, we have used PIP expenditure as a proxy to the actual expenditure under NHM.

11 There are other entities of public health expenditure (like other departments, local bodies, railway, central procurement, ESIS, CGHS etc.) also. However, it has been observed that about 85–90% of the total public expenditure has been made through these two entities [3, 8, 27]. As the other data are not easily available, we have restricted our analysis within the two-major sources of public health expenditure of the states.

12 In short, after cross-classifying the budget and NHM data, we have calculated the total expenditure made by the government in in-patient care in the public institutes. The user fees collected during public sector hospitalization have been calculated from NSS data and both the expenditures have been divided with the total utilization of the public hospitals to calculate per-hospitalization OOP expenditure and subsidy in the public sector. For private sector, total user fee has been calculated from the NSS data. For the limitation of the methodology and other details of it please check Tamil Nadu State Health Accounts 2013–14 [8].

13 However, the pattern and inferences remain same for both the results. It has to be noted here that, the method of benefit incidence only suggests the relative position of the socio-economic classes in terms of subsidy distribution not the actual subsidy amount.
