# Supplementary material for: Health financing strategies to reduce out-of-pocket burden in India: a comparative study of three states
Source: BMC Health Serv Res. 2018 Nov 3;18:830. doi: 10.1186/s12913-018-3633-5 (PMC6215655; doi:10.1186/s12913-018-3633-5)
Supplement: Supplementary file 1 — Illustration of Benefit Incidence Analysis. (DOCX 20 kb) [file 12913_2018_3633_MOESM1_ESM.docx]

**APPENDIX-B**

We have illustrated the benefit incidence analysis method with an example in this section. We have presented four (quartile) MPCE classes – P, LM, UM and R. Those people who have been hospitalized in a public hospital during last 365 days has been considered for the present analysis. In this example, 3 persons from the P (P_1_, P_2_ & P_3_), 2 persons each from LM (LM_1_ & LM_2_) & UM (UM_1_ & UM_2_) and 1 person from R (R_1_) class have been hospitalized (column-2). Therefore, the utilization of (ε_ij_) 4 classes are – 3, 2, 2 & 1 respectively for P, LM, UM and R. The overall utilization by the all groups together (ε_i_) is 8 in the given example. We then calculated the utilization rate (χ_ij_) and represented in column-5. The OOPE is the user charges and/or other medical care related expenditure made by the households (column-6) during utilization of healthcare services.

**Table B1: Estimation of Benefit Incidence Analysis: An Example**

| **MPCE Class**  **(1)** | **Hospitalized member**  **(2)** | **Utilization of the group**  **(3)** | **Overall Utilization**  **(4)** | **Utilization rate**  **(5)** | **OOPE**  **(6)** | **Cost of the service**  **(7)** | **Net subsidy**  **(8)** | **Individual subsidy benefit**  **(9)** | **Group subsidy benefit**  **(10)** | **Benefit Incidence**  **(11)** |
| --- | --- | --- | --- | --- | --- | --- | --- | --- | --- | --- |
| P | P_1_ | 3 | 8 | 3/8 = 0.375 | 240 | 400 | (400-240) = 160 | 60.00 | 180.00 | 52.36% |
|  | P_2_ | 3 | 8 | 3/8 = 0.375 | 200 | 450 | (450-200) = 250 | 93.75 |  |  |
|  | P_3_ | 3 | 8 | 3/8 = 0.375 | 450 | 520 | (520-450) = 70 | 26.25 |  |  |
| LM | LM_1_ | 2 | 8 | 2/8 = 0.25 | 400 | 700 | (700-400) = 300 | 75.00 | 112.50 | 32.73% |
|  | LM_2_ | 2 | 8 | 2/8 = 0.25 | 750 | 900 | (900-750) = 150 | 37.50 |  |  |
| UM | UM_1_ | 2 | 8 | 2/8 = 0.25 | 745 | 850 | (850-745) = 105 | 26.25 | 38.75 | 11.27% |
|  | UM_2_ | 2 | 8 | 2/8 = 0.25 | 500 | 550 | (550-500) = 50 | 12.50 |  |  |
| R | R_1_ | 1 | 8 | 1/8 = 0.125 | 1000 | 1100 | (1100-1000) = 100 | 12.50 | 12.50 | 3.64% |

Note: P-poorest, LM-lower middle, UM-upper middle, R-richest, OOPE-Out-of-pocket expenditure.

Source: Authors’ estimation.

The cost of providing the care to each patient (varies across various quality & other aspects of services) is listed in column-7 and the net subsidy (subsidy after OOPE) has been calculated (μ_i_) in column-8. According to the BIA methodology, the individual level subsidy benefit enjoyed by each patient is the net subsidy weighted by the utilization rate. It has been calculated and presented in column-9. Now, we have added subsidy benefit of each class (Column-10) to get the group subsidy benefit (ψ_j_). Then the percentage share of each class in total subsidy benefit has been calculated and presented in the final column. It has to be noted here that, the theoretical range for utilization rate (χ_ij_) is zero to one as it is a ratio. On the other hand, ψ_j_ could be any positive number starting from zero. In this study, the benefit incidence analysis has been carried out and corresponding result has been reported in Table-6. The above illustration is a simplified version of our estimation; however, in our study we have considered state, sector and diseases along with the MPCE classes to capture the variation of expenditure in treatment across geographical locations and type of ailments.
