# Supplementary material for: Health financing strategies to reduce out-of-pocket burden in India: a comparative study of three states
Source: BMC Health Serv Res. 2018 Nov 3;18:830. doi: 10.1186/s12913-018-3633-5 (PMC6215655; doi:10.1186/s12913-018-3633-5)
Supplement: Supplementary file 2 — Additional tables. (DOCX 59 kb) [file 12913_2018_3633_MOESM2_ESM.docx]

**APPENDIX - A**

| **Table A1: MPCE Class wise Distribution of Ailments in 2014 & 2004** | | | | | | | | | | | | | | | | |
| --- | --- | --- | --- | --- | --- | --- | --- | --- | --- | --- | --- | --- | --- | --- | --- | --- |
| **State** | **Ailment** | **Rural** | | | | | **Urban** | | | | | **Combined** | | | | |
|  |  | **P** | **LM** | **UM** | **R** | **All** | **P** | **LM** | **UM** | **R** | **All** | **P** | **LM** | **UM** | **R** | **All** |
| **TN** | **Com** | 40.7 (29.4) | 30.7 (27.5) | 18.8 (24.5) | 25.9 (24.2) | **28.2 (26.2)** | 35.1 (28.0) | 34.6 (24.5) | 27.7 (15.0) | 22.6 (11.8) | **30.0 (20.4)** | 37.5 (28.8) | 32.8 (26.5) | 23.5 (21.3) | 24.5 (20.4) | **29.2 (24.2)** |
|  | **Non-Com** | 41.6 (32.0) | 42.7 (36.5) | 58.9 (45.0) | 47.7 (42.8) | **48.6 (39.4)** | 44.5 (41.2) | 43.2 (28.8) | 35.3 (46.5) | 57.6 (50.1) | **44.5 (41.6)** | 43.3 (36.0) | 43.0 (34.1) | 46.4 (45.5) | 52.1 (45.1) | **46.4 (40.2)** |
|  | **I & D** | 10.0 (15.7) | 14.7 (12.4) | 15.7 (9.0) | 12.9 (10.1) | **13.3 (11.6)** | 11.0 (9.5) | 15.2 (11.7) | 15.4 (11.4) | 12.9 (12.8) | **13.5 (11.2)** | 10.6 (13.0) | 15.0 (12.2) | 15.5 (9.8) | 12.9 (10.9) | **13.4 (11.4)** |
|  | **Others** | 7.7 (22.9) | 11.8 (23.7) | 6.6 (21.5) | 13.5 (22.9) | **9.9 (22.7)** | 9.4 (21.2) | 7.0 (35.1) | 21.6 (27.1) | 6.8 (25.3) | **12.0 (26.8)** | 8.6 (22.2) | 9.3 (27.2) | 14.6 (23.4) | 10.5 (23.7) | **11.0 (24.2)** |
| **RAJ** | **Com** | 32.4 (30.0) | 28.5 (33.9) | 28.0 (22.8) | 28.5 (36.3) | **29.5 (32.0)** | 28.4 (33.4) | 24.9 (23.0) | 28.3 (29.0) | 25.3 (15.5) | **26.6 (25.5)** | 31.4 (31.0) | 27.0 (31.1) | 28.1 (24.7) | 27.8 (31.7) | **28.6 (30.3)** |
|  | **Non-Com** | 33.0 (19.7) | 46.9 (38.7) | 41.1 (26.5) | 51.2 (22.5) | **42.8 (26.8)** | 48.6 (27.0) | 47.3 (42.9) | 53.1 (40.7) | 43.9 (41.8) | **48.3 (37.2)** | 36.7 (21.9) | 47.0 (39.8) | 44.6 (30.9) | 49.6 (26.8) | **44.4 (29.6)** |
|  | **I & D** | 21.2 (11.6) | 14.1 (11.2) | 18.5 (17.2) | 11.5 (12.0) | **16.4 (12.4)** | 12.4 (14.1) | 17.9 (7.6) | 9.8 (8.9) | 18.6 (10.7) | **14.8 (10.7)** | 19.1 (12.4) | 15.7 (10.3) | 16.0 (14.6) | 13.0 (11.7) | **16.0 (12.0)** |
|  | **Others** | 13.4 (38.8) | 10.5 (16.2) | 12.4 (33.5) | 8.8 (29.3) | **11.3 (28.8)** | 10.6 (25.5) | 10.0 (26.5) | 8.9 (21.4) | 12.2 (31.9) | **10.3 (26.6)** | 12.7 (34.7) | 10.3 (18.8) | 11.4 (29.7) | 9.5 (29.9) | **11.0 (28.2)** |
| **WB** | **Com** | 25.4 (44.6) | 26.3 (31.1) | 31.3 (32.7) | 22.6 (23.6) | **26.6 (32.9)** | 24.2 (24.1) | 20.9 (26.1) | 10.2 (24.4) | 12.1 (17.4) | **16.4 (23.1)** | 25.0 (36.5) | 24.4 (29.1) | 24.2 (31.2) | 18.9 (21.7) | **23.1 (29.6)** |
|  | **Non-Com** | 51.1 (22.8) | 39.4 (21.9) | 33.9 (30.3) | 52.3 (32.2) | **43.8 (26.9)** | 54.1 (29.9) | 59.7 (36.8) | 67.2 (36.4) | 59.3 (55.7) | **60.4 (39.1)** | 52.1 (25.5) | 46.4 (27.9) | 45.2 (31.4) | 54.8 (39.6) | **49.5 (31.0)** |
|  | **I & D** | 16.3 (8.9) | 24.2 (20.8) | 26.4 (11.7) | 15.4 (18.6) | **20.8 (15.0)** | 16.1 (13.8) | 11.9 (6.9) | 14.8 (7.5) | 18.4 (6.3) | **15.3 (9.0)** | 16.3 (10.8) | 20.0 (15.2) | 22.5 (11.0) | 16.4 (14.7) | **18.9 (13.0)** |
|  | **Others** | 7.2 (23.7) | 10.1 (26.2) | 8.4 (25.3) | 9.7 (25.6) | **8.9 (25.2)** | 5.5 (32.3) | 7.5 (30.1) | 7.8 (31.7) | 10.2 (20.6) | **7.8 (28.7)** | 6.6 (27.1) | 9.2 (27.8) | 8.2 (26.4) | 9.9 (24.0) | **8.5 (26.4)** |

Note: Com-Communicable, Non-com-Non-communicable, I&D-Injuries & Disabilities, MCH & CB-Maternal, child health and child birth, P-poorest, LM-lower middle, UM-upper middle, R-richest. Figures in the parenthesis are the reports the share of 2004. – almost same, - decreased, - increased.

Source: Authors’ estimation from NSS 71^st^ and NSS 60^th^ round data.

| **Table A2: Ailment wise Share of Public Institutions in all Hospitalization Cases during 2014 & 2004** | | | | | | | | | | | | | | | | |
| --- | --- | --- | --- | --- | --- | --- | --- | --- | --- | --- | --- | --- | --- | --- | --- | --- |
| **State** | **Ailment** | **Rural** | | | | | **Urban** | | | | | **Combine** | | | | |
|  |  | **P** | **LM** | **UM** | **R** | **All** | **P** | **LM** | **UM** | **R** | **All** | **P** | **LM** | **UM** | **R** | **All** |
| **TN** | **Com** | 59.8 (38.0) | 56.5 (52.5) | 43.9 (41.0) | 23.8 (46.3) | **46.2 (44.8)** | 47.4 (62.0) | 24.1 (48.7) | 34.0 (28.7) | 8.7 (11.2) | **32.4 (45.8)** | 53.1 (48.2) | 38.7 (51.4) | 37.7 (38.1) | 17.6 (39.9) | **38.8 (45.1)** |
|  | **Non-com** | 49.1 (45.4) | 44.9 (46.5) | 27.9 (35.9) | 21.1 (25.9) | **32.8 (37.5)** | 44.4 (63.9) | 28.4 (30.3) | 28.5 (35.4) | 14.2 (15.3) | **29.1 (38.0)** | 46.3 (54.6) | 36.2 (42.3) | 28.2 (35.7) | 17.7 (22.2) | **31.0 (37.6)** |
|  | **I & D** | 67.5 (43.1) | 70.0 (53.7) | 55.7 (50.4) | 37.8 (26.9) | **55.4 (44.2)** | 53.6 (50.8) | 46.9 (47.9) | 16.2 (27.3) | 18.7 (8.0) | **32.0 (33.5)** | 59.2 (45.6) | 57.7 (51.9) | 35.0 (41.3) | 29.3 (20.0) | **43.2 (40.5)** |
|  | **Others** | 54.2 (46.6) | 56.1 (49.0) | 43.6 (36.7) | 15.2 (19.0) | **36.5 (37.8)** | 38.0 (41.2) | 23.5 (32.2) | 7.4 (22.0) | 13.0 (9.4) | **16.9 (27.1)** | 44.1 (44.4) | 43.4 (42.3) | 15.1 (30.9) | 14.6 (15.8) | **25.3 (33.7)** |
|  | **MCH & CB** | 75.4 (65.8) | 76.3 (55.9) | 59.4 (60.8) | 34.6 (23.3) | **66.2 (54.8)** | 69.3 (55.4) | 50.4 (58.6) | 38.2 (40.5) | 15.2 (31.6) | **49.1 (49.5)** | 72.3 (61.8) | 66.1 (56.5) | 48.5 (51.2) | 24.9 (25.5) | **58.0 (53.0)** |
|  | **All** | **61.3 (51.5)** | **60.1 (51.6)** | **40.0 (42.2)** | **24.5 (28.2)** | **45.4 (44.1)** | **51.2 (56.7)** | **33.5 (42.6)** | **25.8 (32.5)** | **13.7 (15.4)** | **32.6 (39.6)** | **55.6 (53.7)** | **47.1 (49.0)** | **32.5 (38.7)** | **19.6 (24.3)** | **38.9 (42.5)** |
| **RAJ** | **Com** | 61.1 (74.1) | 44.1 (58.5) | 53.0 (67.8) | 53.2 (37.3) | **53.9 (55.7)** | 82.6 (79.2) | 69.2 (53.3) | 42.6 (68.2) | 36.4 (57.6) | **59.1 (67.8)** | 65.7 (75.8) | 53.9 (57.5) | 50.0 (67.9) | 49.8 (39.5) | **55.3 (58.4)** |
|  | **Non-com** | 71.5 (59.3) | 61.2 (63.9) | 67.5 (42.8) | 35.1 (43.9) | **55.8 (54.7)** | 69.6 (77.7) | 51.6 (63.2) | 49.2 (69.4) | 42.5 (50.1) | **53.2 (64.1)** | 70.9 (66.2) | 57.1 (63.7) | 61.1 (53.8) | 36.5 (46.1) | **55.0 (57.9)** |
|  | **I & D** | 90.2 (44.0) | 73.4 (46.3) | 21.7 (37.8) | 38.2 (57.6) | **58.2 (47.5)** | 51.2 (75.5) | 48.8 (72.9) | 48.1 (39.3) | 61.4 (43.5) | **52.3 (61.5)** | 84.2 (55.0) | 61.6 (51.2) | 26.4 (38.1) | 45.4 (54.7) | **56.6 (50.8)** |
|  | **Others** | 44.1 (39.0) | 34.5 (63.0) | 53.7 (54.3) | 29.4 (46.8) | **41.7 (47.8)** | 89.6 (72.2) | 38.5 (38.6) | 28.4 (53.7) | 45.6 (64.8) | **49.5 (58.9)** | 53.2 (46.4) | 36.1 (54.4) | 47.9 (54.1) | 33.9 (51.1) | **43.8 (50.6)** |
|  | **MCH & CB** | 88.9 (18.1) | 79.8 (11.5) | 82.3 (19.3) | 65.8 (18.5) | **80.9 (16.5)** | 78.4 (31.9) | 65.5 (26.6) | 52.0 (44.3) | 38.7 (41.1) | **65.0 (34.8)** | 86.4 (20.9) | 75.9 (13.1) | 75.3 (23.5) | 62.5 (22.3) | **77.3 (19.6)** |
|  | **All** | **78.5 (32.8)** | **67.2 (35.9)** | **64.8 (34.1)** | **48.7 (35.7)** | **65.6 (34.5)** | **75.7 (56.2)** | **57.9 (48.0)** | **47.5 (56.6)** | **43.6 (51.7)** | **58.0 (53.5)** | **77.8 (38.6)** | **63.9 (38.1)** | **60.1 (39.5)** | **47.7 (39.0)** | **63.6 (38.7)** |
| **WB** | **Com** | 96.2 (91.5) | 86.3 (92.0) | 93.2 (78.9) | 79.6 (54.3) | **89.4 (81.4)** | 88.4 (88.3) | 71.6 (69.5) | 57.5 (64.0) | 38.3 (26.0) | **68.2 (67.2)** | 93.8 (90.7) | 81.9 (83.8) | 88.1 (76.8) | 70.3 (47.1) | **84.2 (77.7)** |
|  | **Non-com** | 80.2 (94.0) | 67.1 (76.1) | 61.0 (82.0) | 74.8 (54.9) | **71.7 (74.8)** | 59.2 (85.9) | 63.2 (80.4) | 48.8 (80.3) | 25.7 (28.4) | **48.4 (63.7)** | 73.2 (90.3) | 65.3 (78.4) | 54.9 (81.7) | 56.0 (43.2) | **62.0 (70.1)** |
|  | **I & D** | 87.5 (95.8) | 84.6 (94.5) | 83.1 (74.8) | 60.0 (78.8) | **80.1 (85.7)** | 70.0 (85.2) | 84.7 (74.6) | 62.7 (65.0) | 16.4 (29.1) | **54.1 (71.3)** | 81.9 (90.5) | 84.6 (90.8) | 78.6 (73.6) | 42.7 (72.1) | **73.0 (82.3)** |
|  | **Others** | 57.3 (84.8) | 51.8 (77.5) | 62.2 (76.7) | 66.3 (60.9) | **59.5 (74.6)** | 76.2 (83.6) | 55.8 (65.5) | 42.6 (51.4) | 20.6 (37.9) | **43.5 (65.5)** | 62.4 (84.2) | 52.9 (72.2) | 55.9 (71.3) | 49.7 (54.7) | **54.5 (71.3)** |
|  | **MCH & CB** | 82.2 (12.0) | 84.8 (18.3) | 83.1 (30.9) | 63.0 (59.1) | **78.6 (26.1)** | 82.9 (53.6) | 71.5 (25.4) | 50.1 (51.2) | 17.7 (47.1) | **65.7 (40.5)** | 82.4 (19.3) | 79.9 (20.0) | 76.3 (32.1) | 56.5 (57.6) | **75.3 (28.4)** |
|  | **All** | **83.2 (50.7)** | **77.3 (54.0)** | **78.7 (58.2)** | **70.1 (60.2)** | **77.5 (55.5)** | **73.5 (77.5)** | **68.1 (59.2)** | **51.1 (63.4)** | **24.2 (32.4)** | **55.1 (59.9)** | **80.2 (58.7)** | **74.1 (55.7)** | **70.2 (58.9)** | **56.0 (52.9)** | **70.4 (56.6)** |

Note: Com-Communicable, Non-com-Non-communicable, I&D-Injuries & Disabilities, MCH & CB-Maternal, child health and child birth, P-poorest, LM-lower middle, UM-upper middle, R-richest. Figures in the parenthesis are the reports the share of 2004.

Source: Authors’ estimation from NSS 71^st^ and NSS 60^th^ round data.

| **Table A3: MPCE Class wise Estimated Subsidy Share during 2014 (in %)** | | | | | | | |
| --- | --- | --- | --- | --- | --- | --- | --- |
| **Sector** | **MPCE** | **TN** | | **RAJ** | | **WB** | |
|  |  | **Method-I (NSS)** | **Method-II (Budget)** | **Method-I (NSS)** | **Method-II (Budget)** | **Method-I (NSS)** | **Method-II (Budget)** |
| **Rural** | **P** | **42.25** | **34.05** | **44.43** | **32.81** | 25.99 | 25.89 |
|  | **LM** | 26.58 | 27.32 | 15.13 | 18.22 | 19.44 | 24.46 |
|  | **UM** | 20.48 | 23.99 | 20.66 | 23.29 | **28.15** | **30.27** |
|  | **R** | 10.69 | 14.65 | 19.78 | 25.68 | 26.41 | 19.37 |
| **Urban** | **P** | **64.35** | **44.72** | 38.59 | 30.99 | **36.21** | **31.69** |
|  | **LM** | 11.01 | 22.03 | **38.88** | **35.35** | 31.53 | 31.09 |
|  | **UM** | 20.21 | 22.82 | 14.86 | 18.07 | 24.66 | 26.15 |
|  | **R** | 4.43 | 10.42 | 7.66 | 15.59 | 7.60 | 11.08 |
| **Combine** | **P** | **54.17** | **39.47** | **41.53** | **32.28** | **31.30** | 27.80 |
|  | **LM** | 18.18 | 24.63 | 26.94 | 23.21 | 25.72 | 26.64 |
|  | **UM** | 20.33 | 23.40 | 17.78 | 21.77 | 26.34 | **28.92** |
|  | **R** | 7.31 | 12.50 | 13.75 | 22.74 | 16.64 | 16.65 |

Note: P-poorest, LM-lower middle, UM-upper middle, R-richest,

Source: Estimated from NSS 71^st^ round, DDGs (Expenditure budget) and NHM data.

| **Table A4: Concentration Indices of the Subsidy Distribution** | | | | | | | |
| --- | --- | --- | --- | --- | --- | --- | --- |
| **Sector** | **MPCE** | **TN** | | **RAJ** | | **WB** | |
|  |  | **2004** | **2014** | **2004** | **2014** | **2004** | **2014** |
| **Rural** | **P** | 0.0399 | -0.0392 | 0.0411 | -0.0733 | 0.0090 | -0.0164 |
|  | **LM** | -0.0188 | -0.0153 | -0.0488 | 0.0138 | 0.0084 | 0.0218 |
|  | **UM** | -0.0397 | -0.0245 | 0.0314 | -0.0022 | -0.0173 | -0.0043 |
|  | **R** | 0.0000 | 0.0000 | 0.0000 | 0.0000 | 0.0000 | 0.0000 |
|  | **All** | **-0.0185** | **-0.0789** | **0.0238** | **-0.0616** | **0.0001** | **0.0011** |
| **Urban** | **P** | -0.0970 | -0.1334 | -0.0311 | 0.0007 | -0.0012 | -0.0117 |
|  | **LM** | -0.0009 | 0.0230 | -0.0110 | -0.0601 | -0.0859 | -0.0172 |
|  | **UM** | -0.0361 | -0.0395 | 0.0840 | -0.0180 | 0.0152 | -0.0427 |
|  | **R** | 0.0000 | 0.0000 | 0.0000 | 0.0000 | 0.0000 | 0.0000 |
|  | **All** | **-0.1340** | **-0.1498** | **0.0419** | **-0.0773** | **-0.0719** | **-0.0715** |
| **Combine** | **P** | -0.0366 | -0.0900 | -0.0084 | -0.0365 | 0.0046 | -0.0140 |
|  | **LM** | -0.0088 | 0.0054 | -0.0229 | -0.0229 | -0.0328 | 0.0016 |
|  | **UM** | -0.0378 | -0.0326 | 0.0675 | -0.0101 | -0.0031 | -0.0243 |
|  | **R** | 0.0000 | 0.0000 | 0.0000 | 0.0000 | 0.0000 | 0.0000 |
|  | **All** | **-0.0832** | **-0.1172** | **0.0362** | **-0.0695** | **-0.0314** | **-0.0367** |

Note: P-poorest, LM-lower middle, UM-upper middle, R-richest,

Source: Estimated from NSS 60^th^ & 71^st^ round, DDGs (Expenditure budget) and NHM data.

**Table A5: Utilization of Healthcare Services during Public Sector Hospitalization (in %)**

| **Service** | **State** | **Payment** | **2014** | | | | **2004** | | | |
| --- | --- | --- | --- | --- | --- | --- | --- | --- | --- | --- |
|  |  |  | **P** | **LM** | **UM** | **R** | **P** | **LM** | **UM** | **R** |
| **Ward Type** | **TN** | **Free** | 41.50 | 23.26 | 23.30 | 11.94 | 30.91 | 30.16 | 24.56 | 14.37 |
|  |  | **Paying General** | 7.18 | 5.64 | 27.54 | 59.64 | 16.26 | 34.93 | 25.40 | 23.41 |
|  |  | **Paying Special** | 0.00 | 0.00 | 25.73 | 74.27 | 46.46 | 4.65 | 27.94 | 20.95 |
|  | **RAJ** | **Free** | 37.29 | 23.97 | 21.66 | 17.09 | 30.55 | 28.39 | 15.77 | 25.29 |
|  |  | **Paying General** | 45.78 | 22.20 | 17.51 | 14.51 | 31.98 | 38.18 | 11.41 | 18.42 |
|  |  | **Paying Special** | 0.00 | 30.66 | 65.37 | 3.97 | 37.99 | 7.09 | 17.98 | 36.93 |
|  | **WB** | **Free** | 31.22 | 25.32 | 26.31 | 17.15 | 33.87 | 27.64 | 21.97 | 16.52 |
|  |  | **Paying General** | 17.33 | 24.38 | 24.85 | 33.44 | 26.95 | 33.63 | 14.74 | 24.68 |
|  |  | **Paying Special** | 10.10 | 35.37 | 24.98 | 29.56 | 4.32 | 47.95 | 17.18 | 30.55 |
| **Surgery** | **TN** | **Not received/required** | 41.76 | 24.41 | 22.66 | 11.17 | 32.96 | 29.91 | 23.02 | 14.12 |
|  |  | **Free** | 39.90 | 18.86 | 25.66 | 15.58 | 21.76 | 32.36 | 30.19 | 15.70 |
|  |  | **Partly free** | 0.00 | 100.00 | 0.00 | 0.00 | 53.96 | 7.25 | 30.44 | 8.35 |
|  |  | **On payment** | 88.42 | 11.58 | 0.00 | 0.00 | 35.12 | 10.40 | 16.76 | 37.73 |
|  | **RAJ** | **Not received/required** | 38.05 | 24.28 | 21.72 | 15.94 | 32.09 | 29.20 | 15.68 | 23.04 |
|  |  | **Free** | 35.13 | 22.71 | 22.11 | 20.05 | 26.82 | 27.64 | 15.74 | 29.80 |
|  |  | **Partly free** | 11.33 | 39.29 | 30.10 | 19.29 | 4.53 | 37.33 | 10.05 | 48.09 |
|  |  | **On payment** | 27.65 | 15.55 | 24.42 | 32.37 | 24.11 | 17.74 | 13.78 | 44.36 |
|  | **WB** | **Not received/required** | 28.05 | 25.41 | 26.77 | 19.78 | 33.36 | 28.14 | 22.15 | 16.34 |
|  |  | **Free** | 40.92 | 24.45 | 24.65 | 9.99 | 29.04 | 31.07 | 13.92 | 25.96 |
|  |  | **Partly free** | 23.55 | 14.04 | 24.91 | 37.51 | 54.93 | 15.50 | 11.45 | 18.12 |
|  |  | **On payment** | 22.48 | 27.81 | 21.31 | 28.40 | 27.00 | 32.65 | 16.97 | 23.38 |
| **Medicine** | **TN** | **Not received/required** | 76.22 | 0.00 | 20.22 | 3.56 | 0.00 | 0.00 | 0.00 | 0.00 |
|  |  | **Free** | 42.27 | 23.79 | 22.20 | 11.75 | 32.47 | 29.96 | 25.09 | 12.47 |
|  |  | **Partly free** | 33.16 | 20.74 | 28.64 | 17.45 | 26.75 | 34.46 | 19.01 | 19.77 |
|  |  | **On payment** | 26.14 | 8.58 | 55.95 | 9.32 | 9.70 | 20.08 | 35.10 | 35.12 |
|  | **RAJ** | **Not received/required** | 16.11 | 14.30 | 16.98 | 52.61 | 0.00 | 68.00 | 0.00 | 32.00 |
|  |  | **Free** | 44.23 | 21.26 | 19.61 | 14.90 | 33.93 | 24.20 | 16.92 | 24.95 |
|  |  | **Partly free** | 27.10 | 30.03 | 26.13 | 16.73 | 23.63 | 20.68 | 9.42 | 46.28 |
|  |  | **On payment** | 39.57 | 16.41 | 19.73 | 24.30 | 31.90 | 29.98 | 16.52 | 21.60 |
|  | **WB** | **Not received/required** | 5.79 | 7.82 | 18.28 | 68.12 | 30.09 | 13.05 | 49.18 | 7.69 |
|  |  | **Free** | 35.98 | 18.78 | 32.35 | 12.88 | 37.95 | 24.23 | 19.03 | 18.79 |
|  |  | **Partly free** | 29.83 | 27.04 | 28.03 | 15.11 | 33.34 | 31.48 | 19.21 | 15.98 |
|  |  | **On payment** | 28.94 | 25.98 | 22.36 | 22.72 | 30.99 | 26.78 | 21.86 | 20.36 |
| **X-ray/ECG/EEG/Scan** | **TN** | **Not received/required** | 45.20 | 24.50 | 22.93 | 7.37 | 28.49 | 32.72 | 22.52 | 16.27 |
|  |  | **Free** | 41.32 | 22.26 | 23.42 | 13.00 | 34.02 | 28.63 | 25.47 | 11.88 |
|  |  | **Partly free** | 10.64 | 32.10 | 31.64 | 25.61 | 25.32 | 37.51 | 31.12 | 6.05 |
|  |  | **On payment** | 44.76 | 21.02 | 19.35 | 14.87 | 20.63 | 22.28 | 29.64 | 27.46 |
|  | **RAJ** | **Not received/required** | 42.36 | 23.07 | 17.87 | 16.70 | 35.32 | 24.74 | 18.76 | 21.18 |
|  |  | **Free** | 35.46 | 22.25 | 25.16 | 17.12 | 20.05 | 29.15 | 15.87 | 34.93 |
|  |  | **Partly free** | 15.24 | 40.93 | 27.56 | 16.27 | 36.80 | 15.68 | 7.44 | 40.08 |
|  |  | **On payment** | 45.46 | 17.29 | 19.23 | 18.02 | 28.94 | 31.47 | 14.28 | 25.31 |
|  | **WB** | **Not received/required** | 31.19 | 24.86 | 28.18 | 15.78 | 36.68 | 27.93 | 23.32 | 12.08 |
|  |  | **Free** | 28.99 | 21.91 | 22.06 | 27.04 | 33.22 | 34.41 | 12.74 | 19.63 |
|  |  | **Partly free** | 33.62 | 27.16 | 24.20 | 15.02 | 21.62 | 27.91 | 26.99 | 23.49 |
|  |  | **On payment** | 26.25 | 27.16 | 24.90 | 21.69 | 26.77 | 27.55 | 18.11 | 27.58 |
| **Other Diagnostic Tests** | **TN** | **Not received/required** | 41.15 | 23.20 | 23.67 | 11.98 | 31.10 | 31.03 | 25.99 | 11.88 |
|  |  | **Free** | 43.54 | 22.89 | 21.51 | 12.07 | 31.37 | 31.00 | 23.39 | 14.25 |
|  |  | **Partly free** | 11.14 | 29.13 | 45.96 | 13.76 | 26.96 | 27.98 | 33.86 | 11.20 |
|  |  | **On payment** | 37.56 | 19.95 | 30.06 | 12.43 | 12.89 | 11.90 | 37.69 | 37.52 |
|  | **RAJ** | **Not received/required** | 38.22 | 25.67 | 16.52 | 19.59 | 40.26 | 26.66 | 13.45 | 19.63 |
|  |  | **Free** | 40.21 | 21.83 | 22.73 | 15.23 | 22.58 | 41.53 | 15.34 | 20.55 |
|  |  | **Partly free** | 19.82 | 38.07 | 26.44 | 15.68 | 35.80 | 10.13 | 9.52 | 44.55 |
|  |  | **On payment** | 35.45 | 14.92 | 25.38 | 24.25 | 27.84 | 29.59 | 17.12 | 25.44 |
|  | **WB** | **Not received/required** | 29.03 | 25.78 | 28.86 | 16.32 | 37.98 | 29.54 | 20.63 | 11.85 |
|  |  | **Free** | 38.40 | 23.71 | 20.70 | 17.18 | 30.92 | 27.05 | 22.43 | 19.60 |
|  |  | **Partly free** | 28.79 | 27.20 | 23.73 | 20.28 | 23.22 | 35.91 | 20.10 | 20.77 |
|  |  | **On payment** | 26.65 | 25.01 | 26.76 | 21.58 | 28.07 | 27.23 | 20.56 | 24.13 |

Note: P-Poorest, LM-Lower-middle, UM-Upper-middle, R-Rich.

Source: Authors’ estimation from NSS 71^st^ & 60^th^ round data.
